# Current Evidence about Developmental Dysplasia of the Hip in Pregnancy

**DOI:** 10.3390/medicina57070655

**Published:** 2021-06-26

**Authors:** Anca Angela Simionescu, Monica Mihaela Cirstoiu, Catalin Cirstoiu, Ana Maria Alexandra Stanescu, Bogdan Crețu

**Affiliations:** 1 Department of Obstetrics and Gynecology, “Carol Davila” University of Medicine and Pharmacy, Filantropia Clinical Hospital, 011171 Bucharest, Romania; asimion2002@yahoo.com; 2 Department of Obstetrics and Gynecology, “Carol Davila” University of Medicine and Pharmacy, University Emergency Hospital, 050098 Bucharest, Romania; dr_cirstoiumonica@yahoo.com; 3 Department of Orthopedics and Traumatology, “Carol Davila” University of Medicine and Pharmacy, University Emergency Hospital, 050098 Bucharest, Romania; catalin.cirstoiu@umfcd.ro (C.C.); jfrbogdan@yahoo.com (B.C.); 4 Department of Family Medicine, “Carol Davila” University of Medicine and Pharmacy, 050474 Bucharest, Romania

**Keywords:** pregnancy, hip dysplasia, pelvic osteotomies

## Abstract

In adults, developmental dysplasia of the hip (DDH) represents a spectrum of disorders. It is commonly found in women in routine orthopedic practice. Hip dysplasia is a leading precursor of joint laxity; when untreated, it can contribute to chronic modifications, such as thickening of the pulvinar and ligamentum teres (which can also elongate), hypertrophy of the transverse acetabular ligament, and osteoarthritis. DDH is presumed to be associated with alterations in pelvic morphology that may affect vaginal birth by the reduction in the transverse diameter of the pelvic inlet or outlet. Here, we provide an overview of the current knowledge of pregnancy-associated DDH. We primarily focused on how a surgical DDH treatment might influence the pelvic shape and size and the effects on the mechanism of birth. We presented the female pelvis from the standpoint of bone and ligament morphology relative to a pelvic osteotomy. Then, we described whether the pregnancy was impacted by previous surgical DDH treatments, performed from infancy to adulthood. In conclusion, hip dysplasia is not associated with high-risk complications during pregnancy or with increased difficulty in vaginal delivery.

## 1. Introduction

In adults, developmental dysplasia of the hip (DDH) represents a common disorder in routine orthopedic practice. The prevalence in adults varies between 0.1% and 12.8%, and it occurs 2.76-fold more frequently in women than in men [1,2,3,4]. Although some immature hips may resolve spontaneously, in adults, untreated DDH can lead to early hip degenerative modifications, instability, limb shortening, cartilage reduction, postural scoliosis, difficulty walking, and chronic back pain [5,6,7]. DDH manifests in a spectrum of anatomical abnormalities, due to the variability in acetabular morphology. An anterior or anterolateral deficiency of the acetabulum, common among females, may lead to excessive acetabular anteversion, hip instability, and hip dysplasia.

Recognized since the times of Hippocrates, by the late 1980s, the spectrum of hip dysplasia at birth (congenital dysplasia of the hip) was well-defined. This spectrum includes subluxation (partial dislocation) of the femoral head, acetabular dysplasia, and complete dislocation of the femoral head from the true acetabulum. However, despite routine screening for hip dysplasia at birth and during infancy, many cases are not diagnosed until adulthood. Hip dysplasia is a leading precursor of joint laxity [8] and chronic modifications, particularly osteoarthritis [9]. DDH is associated with osteoarthritis of the hip in 20–40% of patients [10,11]. Considered a preventable disease, an early diagnosis of hip dysplasia in infants is strongly desirable, because a late diagnosis increases the treatment costs and disease complexity [12]. Hip dysplasia may lead to subluxation and gradual dislocation. Other chronic modifications occur secondarily, such as an obstruction to reduction—caused by thickening of the pulvinar and ligamentum teres, elongation of the ligamentum teres, or hypertrophy of the transverse acetabular ligament, iliopsoas, and hip capsule—or anatomic changes, such as an increase in acetabular or femoral anteversion, flattening of the femoral head, an increase in the obliqueness and a reduction in the concavity of the acetabular roof, or thickening of the medial acetabular wall [13,14].

Some authors consider DDH at birth and DDH in early adulthood two different entities. Adult DDH occurs when the hip was stable in infancy, but hip pain occurs in adolescence or early adulthood. It is debatable whether adult DDH represents a milder variant of infantile DDH that escaped detection at birth, or whether it is a different type of hip disease [15].

The reported prevalence of DDH at birth varies from 1.9% to 30% of live-births, depending on the case definition and ethnicity [16,17,18]. Female sex is a risk factor for joint laxity [19,20], due to the fact that females exhibit reduced hip muscle activity compared to men [21,22]. The incidence of DDH was reduced in preterm infants born at less than 36 weeks of gestation [23]. That finding was counter-intuitive, explained by the enhanced maturity of the hip in premature infants [24,25]. DDH mostly occurs unilaterally (nearly 65% of cases) and on the left side of the body (64.0%) [17], because in the womb, the left leg of the fetus is in an adducted position against the mother’s sacrum [15,26].

Among fertile women 15–49 years old, DDH is presumed to be associated with alterations in pelvic morphology that lead to a reduction in the transverse diameter of the pelvic inlet or outlet [27,28]. Therefore, we investigated the importance of a DDH diagnosis during pregnancy. Here, we provide an overview of the current knowledge of pregnancy-associated DDH. We primarily focused on whether surgical DDH treatment had an obstetric influence on the pelvic shape and size and whether that influence affected the mechanism of birth. The secondary aim was to present etiological factors and the impact of previous surgical DDH treatments on the course of pregnancy.

## 2. Classifications of the Female Pelvis, Based on Bone and Ligament Morphology, and Their Relationships to Pelvic Osteotomy

### 2.1. Pelvis Morphology

**Figure 1:**
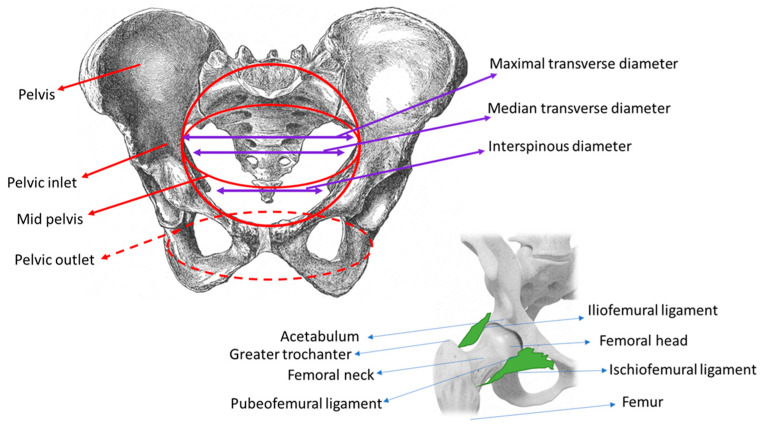
Pelvic girdle and connections through hip-joint articulations.

The pelvic girdle is formed by the articulation of the two coxal bones (right and left) with the sacrum bone through the sacro-spinous and sacro-tuberous ligaments. Anteriorly, the hip bones meet to form the pubic symphysis. Posteriorly, the hip bones unite with the sacrum to form the sacroiliac joints. Obstetricians use three imaginary planes to manage labor: the pelvic inlet, the mid pelvis, including two planes—one at the largest and one at the smallest diameter—and the pelvic outlet (Figure 1).

During labor, the fetus undergoes a series of changes in position, attitude, and presentation to occupy the least amount of space in the intrauterine cavity. In preparation for its entry into the pelvic inlet, the fetal head diameters (i.e., in flexed cephalic presentation: the suboccipitobregmatic and biparietal diameters) and shoulder diameters (biacromial) align with the transverse diameters of the pelvic inlet. The pelvic inlet is defined by the promontory, the anterior edge of the sacral wing, the sacroiliac symphysis, the unnominate line, the eminence of the iliopectineal line, the pectineal ridge, the upper edge of the pubic body, and the pubic symphysis. The most important obstetric diameters are related to the hip joints (Table 1) [29].

Based mainly on the shape of the pelvic inlet, there are four classic pelvic morphologies: gynecoid, android, anthropoid, and platypelloid. Additional morphologies can occur with combinations of these forms. The gynecoid pelvis is observed most frequently, and it is most suitable for a vaginal birth. The anatomic diameters of the gynecoid pelvis are presented in Table 2 [30,31].

### 2.2. Pelvic Osteotomy

The pelvic osteotomy is a surgical procedure for treating acetabular dysplasia. It alters the shape and depth of the bony cup that houses the femoral joint. Various osteotomies have been described, and the choice is based on patient age, the DDH stage, and the scope of the surgical correction. It is recognized that, when an osteotomy is performed in childhood, a remodeling process occurs that reconstructs the shape and structure of the pelvic bone [32]. Osteotomies are classified as reconstructive (e.g., the Salter, Triple osteotomy Steel, Carlioz and Tönnis, Ganz, or Pemberton type) or salvage (e.g., the Shelf or Chiari type). For children under 7 years old, the most common procedures are the Salter innominate osteotomy and the Pemberton osteotomy [33].

**Figure 2:**
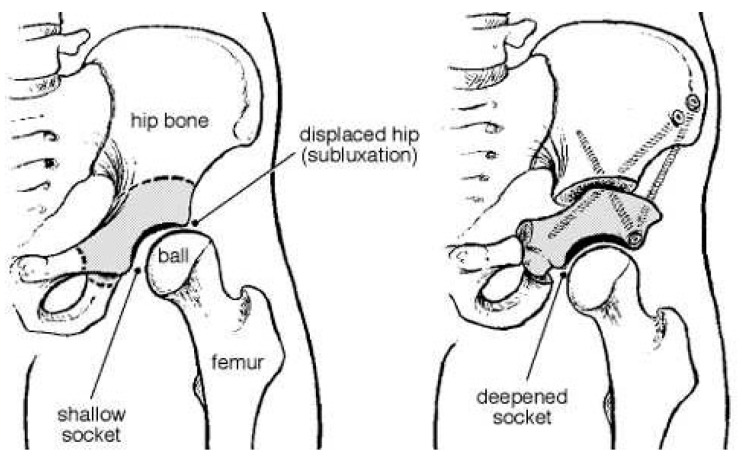
The PAO osteotomy—multiplane reorientation of the acetabulum.

**Figure 3:**
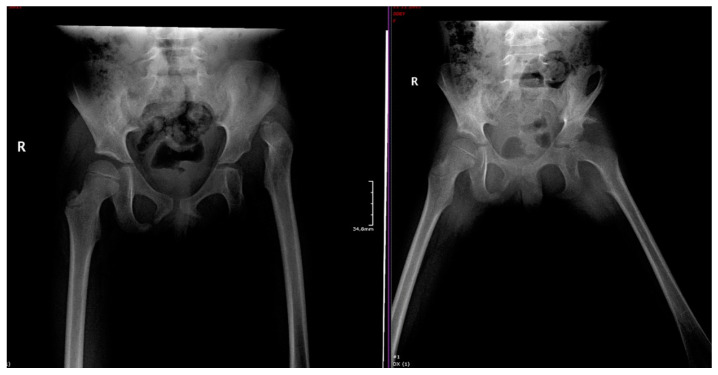
Proper acetabular orientation after a Salter osteotomy in a 5-year-old child. (**Left**) Pre-operative X-ray of the pelvis shows abnormal position of the femur head on the left side. (**Right**) Post-operative X-ray shows reorientation with good femoral head coverage and proper positioning of the bone graft. R: right side of patient; scale: 34.8 mm.

A periacetabular osteotomy (PAO) is a reconstructive osteotomy that allows multiplane reorientation. A PAO does not alter the true diameter of the pelvis or the posterior pillar [34,35] (Figure 2). A Salter osteotomy is a complete trans-iliac osteotomy that allows the entire acetabulum to be redirected and covered (Figure 3). A Pemberton acetabuloplasty is an incomplete osteotomy that allows the shape of the acetabulum to be modified, by hinging the horizontal branch of the triradiate cartilage.

### 2.3. Effects of a Pelvic Osteotomy on the Birth Mechanism

A pelvic osteotomy is an important option for treating symptomatic DDH in young adult women of childbearing age [36,37]. In teenagers and adults with painful hip dysplasia, a Ganz osteotomy is frequently performed. However, a combined procedure may be indicated for this situation. For example, an open reduction might be combined with a femoral-shortening osteotomy and an acetabular procedure. However, after surgery, a re-dislocation and premature triradiate cartilage closure of the hip might occur. These events could lead to changes in pelvic morphology. An incorrect pelvic osteotomy could alter the geometry of the pelvic inlet or outlet and lead to complications during pregnancy or childbirth [32,38,39,40].

A few studies with small numbers of patients and theoretical models have described the influence of pelvic osteotomies on pelvic morphology and the birth canal [32,38,41]. Those studies showed that pelvic osteotomies had no effect on the pelvic inlet, but caused narrowing of the pelvic outlet, particularly after the Salter, Sutherland, and Steel osteotomies. Post-surgical changes can also have clinical consequences, particularly in a non-gynecoid pelvis. Thus, adequate obstetric evaluations are required in case of a medical history of osteotomy.

## 3. Hip Dysplasia at Birth: From Diagnosis to Appropriate Treatment

Screening programs for DDH are a debatable topic [42,43]. They include a clinical examination at birth, an ultrasound hip examination in the first 3 months (universal or targeted to high-risk groups), or a combination of the two. Late DDH detection was found to increase treatment costs significantly [44]. In Romania, clinical screenings at birth by neonatologists and universal ultrasounds are recommended, but there is no national screening program.

The main risk factors for DDH were found to be a family history, breech presentation, and female sex. Additionally, DDH was associated with the first-born infant, left-side hip dysplasia, and the mode of delivery [16].

A family history of DDH increases the risk of developing DDH. First-degree relatives of DDH had a 12-fold higher risk of developing a DDH, while second-grade relatives had a 1.7-fold risk [43,45].

A breech presentation occurs more frequently with female infants than with male infants [46]. A meta-analysis of 20,196 newborns showed that the relative risk (RR) of DDH was 3.75 (95% confidence interval (CI): 2.25–6.24) for breech presentations, compared to non-breech presentations, the RR was 1.39 (95% CI: 1.23–1.57) for family history, compared to no family history, and the RR was 2.54 (95% CI: 2.11–3.05) for newborn females compared to newborn males [16]. The risk was also higher for the first-born infant, compared to subsequent infants, with an RR of 1.44 (95% CI: 1.12–1.86) [16]. Moreover, many studies reported that the occurrence of DDH was significantly higher on the left side than on the right side of the body [47,48,49,50].

Other risk factors for DDH were reported to be associated with specific circumstances, such as: oligohydramnios, the presence of torticollis, and a foot deformity. Many genetic syndromes (e.g., Beukes hip dysplasia) and cerebral palsy were associated with DDH [51]. However, a systematic literature review of population-based studies revealed that only 10–27% of all infants diagnosed with DDH had the identified risk factors [52].

It has often been reported that DDH is hereditary in families [53,54,55]. A genetic predisposition for DDH with an autosomal-dominant transmission mode was linked to chromosomes 4q35, 13q22, and 17q21. More than 25 genes have been associated with DDH, frequently the *HOX*, *TENM3*, and *PAPPA2* genes. Currently, genes implicated in chondrogenesis, chondrocyte differentiation, osteogenesis, and ligament and bone formation are under investigation [56,57,58,59,60]. Additionally, genes such as GDF5 rs143383 and rs143384 were reported by European authors to be associated with DDH TENM3, and HOX genes were more studied outside of Europe [53]. Rouault et al. even reported no association of HOXB9 and COL1A1 in the French population [61].

In a recent meta-analysis, Kenanidis et al. founded specific gene polymorphisms (SNPs) associated with the severity of DDH. The following SNPs: rs143383 of GDF5 gene rs2303486 of HOXB9 gene, and rs3744448 of the Tbx4 gene, and homozygosity for the mutant Taq I Vitamin D receptor t allele and Pvu II pp estrogen receptor genotype, were founded in severe forms of the disease [62].

In a clinical examination conducted at birth, DDH can be diagnosed based on a limb-length discrepancy, laxity, hip immaturity, and severe dysplasia in the hip. In an arthrogram, a neolimbus—a margin of hyaline cartilage—can be found at the ridge of the acetabulum [63]. Other signs are revealed with specific tests. For example, when the Ortolani and Barlow maneuvers are performed at birth and in the neonatal period [64], the femoral head passes over the acetabular ridge [55]. In normal hips, stability is increased by the everted labrum. However, the test is considered positive when a ‘clunk’ (or instability) is felt as the femoral head dislocates (Barlow maneuver) or relocates (Ortolani maneuver). However, clicks felt during the clinical examination have no clinical significance [65].

A clinical examination may be combined with an ultrasound hip evaluation at 6 weeks of life to support the diagnosis and to diagnose subtler signs of dysplastic hips at birth [6,66,67]. However, a Cochrane review concluded that no study has definitively demonstrated that either a universal or a targeted ultrasound strategy could improve the clinical outcome, including late-diagnosed DDH and surgery [67]. Thus, the prescription was ultimately left to the discretion of the attending physician. Infants with positive ultrasound findings are treated with a Pavlik harness, which prevents extension and adduction of the hip joint [68]. In randomized trials, universal ultrasound screening versus clinical examination has not proved its high-quality and utility in reducing the incidence of late cases of dysplasia [43,69,70]. Still, screening practices in many countries are similar. However, the studied parameters (related to the late occurrence of DDH, to ultrasonographic parameters, to experience of ultra-sonographers) may differ, making the comparison between screening programs irrelevant.

It is thus even more important that a thorough screening program is needed, adding genetic testing for familial cases [71].

Radiographs are typically acquired after the age of 3 months, when the ossification of the proximal femoral epiphysis is complete. Dislocated or dislocatable newborn hips that were identified and treated in the neonatal period more often showed normal growth, radiologically, and required less surgical intervention than those diagnosed and treated later in life. In 1879, Roser proposed a prevention program [72] which included the early diagnosis of DDH in the newborn and a fixation of the child’s hip in abduction. Subsequently, Roser’s proposals were supported by the successful results reported by Froelich in 1906, Le Danamy in 1911, and Putti in 1929 [73].

DDH treatment varies with age. Before 3 months old, the Pavlik harness is the primary choice. When that fails, a closed reduction and spica cast are applied in infants 6 to 18 months old, and after 18 months old, an open reduction and spica cast are indicated. After 2 years of age, DDH is treated with an open reduction and a femoral osteotomy or pelvic osteotomy [74,75,76,77]. DDH must be treated early to achieve the best outcomes. The goal of treatment is to obtain a congruent, reduced hip in a well-covered acetabulum. Surgeries—such as an open reduction or femoral osteotomy—would not change the shape of the pelvis; indeed, the pelvis is normal in size and shape for most women with hip dysplasia.

Pelvic surgery performed in childhood has shown high potential for remodeling. Any surgery prior to the age of six years can achieve full recovery, and in adulthood, the patient can accommodate a natural pregnancy and childbirth. When pelvic surgery is performed after six years of age, the pelvic bones may be modified and cause birth complications. In these cases, it would be useful to know what type of surgery was performed on the pelvis. The pelvic diameter would not be limited by a PAO. However, the less common types of pelvic surgery might cause concern, including the Steel, Chiari, Sutherland, and Salter osteotomies, when performed on both hips or after the age of six years. The Salter, Dega, and other types of surgery are performed in young children, and thus, they should not present problems.

Left untreated, DDH can lead to various scenarios, including normal development, hip subluxation, a completely dislocated hip, and a reduced hip with acetabular dysplasia. The evolution of DDH is unknown. Therefore, all DDHs are treated. The long-term evolution depends on the presence or absence of bilateral false acetabulums and hip congruency.

## 4. Management of Pregnancy Associated with Maternal DDH

The management of pregnancy-associated hip dysplasia first requires recognition of the condition. Management also requires a multidisciplinary team, which includes an obstetrician, orthopedic surgeon, general practitioner, midwife, anesthetist, and physiotherapist.

Pregnancy is a physiological state characterized by hormonal, metabolic, vascular, and postural changes that are likely to give rise to joint laxity and a wide variety of musculoskeletal problems. The enlargement of the uterus combined with maternal weight gain modifies the body’s center of gravity and applies mechanical stress on the articulations [78,79], including the acetabulofemoral joint (hip joint). The capsular ligaments in the hip joints (iliofemoral, ischiofemoral, and pubofemoral) are essential for joint stability, static posture, and functional mobility. The iliofemoral ligament reinforces the capsule during external rotation and extension. The ischiofemoral ligament reinforces the capsule during internal rotation in neutral positions and in combined flexion-adduction positions. The pubofemoral ligament restricts excessive abduction and external rotation during hip extension [80]. The labrum and the ligamentum teres serve as secondary restraints in wider external rotations. The structures of the capsule, the labrum, and the zona orbicularis are crucial for rotational and hip stability in distraction [81,82].

Pregnancy is also associated with reductions in the bone mineral status of the whole body and in the hip region [83], and rarely, with transient osteoporosis of the hip [84]. The estimated prevalence of pregnancy-related pelvic girdle pain was about 20% [85]. In addition, during pregnancy, joint pain and the sensation of stiffness and aching in the hips, elbows, knees, fingers, and ankles are common—particularly in the third trimester. A study of 72 pregnant women found a relationship between narrow bilateral ischial tuberosity diameters and a high score in pregnancy-related sacroiliac joint pain during different activities [86].

The clinical presentation of acetabular dysplasia can vary. In some cases, the patient has a known history of DDH that developed in childhood and was treated. In other cases, the clinical signs and symptoms can be subtle. The European Guidelines for the Diagnosis and Treatment of Pelvic Girdle Pain have recommended inspections of walking, posture, and pelvic tilt, palpation of ligaments and muscles, tests for a locked sacroiliac joint, and pain provocation tests for the sacroiliac joint and the pubic symphysis [85]. These recommendations are feasible to perform in the first trimester of pregnancy, but they are very difficult to perform in the third trimester or during labor. The most common symptoms that merit attention are groin pain and a trendelenburg gait and limp pattern, when the pelvis is dropped on to the contralateral side while walking. Pain provocation tests of the sacroiliac joint—particularly Patrick’s test (also known as the Faber test, where pressure is applied after flexion, abduction, and external rotation of the leg, with the patient in the supine position) and Menell’s test (pressure applied to the tuber ischiadicum, with the patient in the prone position, while extending the leg)—have high sensitivity and specificity for problems with hip articulation.

The first type of DDH includes frank hip dysplasia, defined as a lateral center-edge angle (LCEA) ˂ 20°, and borderline hip dysplasia, which is most often defined as an LCEA of 20–25°. The LCEA measures the degree of lateral acetabular bony coverage [15,87,88]. This measurement requires X-ray imaging. Although this imaging exposes the fetus to cumulative doses below 100 mGy—which are not associated with deterministic effects—hip radiography and magnetic resonance imaging should not be used until after birth [89]. Moreover, hip ultrasound imaging during pregnancy provides very inconclusive and unusual results.

A second type of DDH occurs after a pelvic osteotomy. Most patients that undergo a pelvic osteotomy are young women of childbearing age. Thus, the question arises whether an unsuccessful pelvic osteotomy could potentially lead to childbearing and delivery complications. It is important to assess the diameter of the true pelvis in an orthopedic evaluation prior to deciding on the type of delivery.

The standard postoperative evaluation after a pelvic osteotomy includes a clinical evaluation that assesses the mobility, gait, and associated pain. This evaluation is typically followed by a radiographic evaluation, with X-rays and computed tomography.

All studies have shown that pregnancy-associated hip dysplasia was uneventful. The conclusions were unanimous that a cesarean section was not systematically indicated. Fluckiger et al. found that a PAO did not influence the inner anatomy of the pelvis or the birth canal; consequently, a natural birth was performed without complications. The following measurements, averaged over 17 women, showed no significant changes after a PAO: the pelvic entrance (pre-PAO: 15.4 cm, post-PAO: 15.7 cm), the mid-pelvis (pre-PAO: 11.8 cm, post-PAO: 11.8 cm), and the pelvic outlet (pre-PAO: 14.2 cm, post-PAO: 13.7 cm) [41]. Valenzuela et al. found that pelvic pain during pregnancy occurred due to the decrease in range of motion that occurred when the medialization of the hip center of rotation was less than 5 mm [90].

The third type of DDH occurs during pregnancy, in women with uni- or bi-lateral hip prostheses after a total hip arthroplasty, due to previous inflammatory arthritis, osteonecrosis, or congenital hip dysplasia. Sierra et al. analyzed 47 patients and found that hip prostheses had not dislocated, fractured, or loosened during pregnancy or childbirth. 

However, in women with hip prostheses, precautions should be taken during a vaginal delivery. The hip should be maintained in flexion at 90°, and internal rotation should be limited to ensure that the joint is positioned as close to neutral as possible [91].

## 5. Conclusions

Patients with DDH can experience successful pregnancies and childbirth. Orthopedic alliances or screening programs in countries could and should work on a guideline for DDH screening which could make all diagnoses and treatments faster. It can further affect patient care and lessen the debilitating effect on patients and also the economic burden it causes. Hip dysplasia alone is not associated with high-risk pregnancies, complications, or increased difficulty in vaginal deliveries. During pregnancy, it is necessary to check the medical history, and when clinical symptoms are suggestive of DDH, a multidisciplinary team should be consulted. Before labor, a clinical evaluation of the pelvis must be performed, including hip mobility testing combined with a fetal ultrasound evaluation to determine the presentation and biometric parameters. When the pelvis is amenable to a vaginal delivery, there are no particular precautions, other than to position the patient appropriately during the second stage of labor. The need for a caesarean section is rarely influenced by hip dysplasia or a previous surgery for hip dysplasia. For all these reasons, physicians must be correctly informed about hip dysplasia and must provide adequate information to the family.

## Figures and Tables

**Table 1:** The most important pelvic diameters in obstetrics, in relation to hip joints.

Antero-Posterior Diameters	Transverse Diameters	Oblique Diameters
Pelvic Inlet Promonto-suprapubic diameter (true conjugate, conjugate vera, anatomic conjugate): 11.5 cm Promonto-pubic diameter (obstetric conjugate): 10.8–11 cm	Maximal transverse diameter, at the widest point between the unnominate lines: 13.5 cm Median transverse diameter: 13 cm	Oblique diameters right and left: 12 cm
Mid-Pelvis Antero-posterior diameter, from S4 to S5 to the lower border of the pubic symphysis: 11.5 cm	Bispinous: 10.5 cm	Oblique diameters right and left: 11 cm
Pelvic Outlet Antero-posterior diameter from the coccyx to the subpubic area—during the second stage of labor, this diameter is 9.5 cm; by mobilizing the coccyx posteriorly, the diameter reaches 12 cm	Transverse diameter between ischiatic tuberosities: 11 cm	Oblique diameters right and left: 11 cm

As the fetus progresses through the pelvis and birth canal, it must rotate and flex to adapt to the changing shape of the canal. The canal tends to deepen sagittally at its midpoint (midplane), and it often enlarges transversely at the outlet.

**Table 2:** The main diameters of the gynecoid pelvis.

Pelvic inlet	obstetric conjugate > 10.5 cm transverse diameter > 13 cm posterior sagittal diameter > 4.5 cm
Mid-pelvis	Plane of greatest diameter: anteroposterior diameter > 12.5 cm transverse diameter > 12.5 cm, posterior sagittal diameter > 4.5 cm Plane of least diameter: anteroposterior diameter > 12 cm interspinous diameter > 10.5 cm posterior sagittal diameter > 4.5 cm
Outlet pelvis	anteroposterior diameter > 11 cm intertuberous diameter > 11 cm posterior sagittal diameter > 4 cm

## Data Availability

Not applicable.
